# Survival and level of care among breast cancer patients with brain metastases treated with whole brain radiotherapy

**DOI:** 10.1007/s10549-017-4466-3

**Published:** 2017-08-22

**Authors:** Gabriella Frisk, Beatrice Tinge, Sara Ekberg, Sandra Eloranta, L. Magnus Bäcklund, Elisabet Lidbrink, Karin E. Smedby

**Affiliations:** 1 Department of Medicine Solna, Clinical Epidemiology Unit, Karolinska Institute Solna, Karolinska University Hospital, 171 76 Stockholm, Sweden; 2 Department of Medicine Solna, Unit for Experimental Cardiovascular Research, Karolinska Institute Solna, 171 76 Stockholm, Sweden; 3 Department of Oncology-Pathology, Karolinska Institute, Karolinska University Hospital Solna, 171 76 Stockholm, Sweden

**Keywords:** Breast cancer, Brain metastases, Whole brain radiotherapy, Level of care

## Abstract

**Purpose:**

The benefit of whole brain radiotherapy (WBRT) for late stage breast cancer patients with brain metastases has been questioned. In this study we evaluated survival and level of care (hospital or home) following WBRT in a population-based cohort by personal and tumor characteristics.

**Methods:**

We identified 241 consecutive patients with breast cancer and brain metastases receiving WBRT in Stockholm, Sweden, 1999–2012. Through review of medical records, we collected data on prognostic determinants including level of care before and after WBRT. Survival was estimated using Cox regression, and odds ratios (OR) of not coming home using logistic regression.

**Results:**

Median age at WBRT was 58 years (range 30--–88 years). Most patients (*n* = 212, 88%) were treated with 4 Gray × 5. Median survival following WBRT was 2.9 months (interquartile range 1.1–6.6 months), and 57 patients (24%) were never discharged from hospital. Poor performance status and triple-negative tumors were associated with short survival (WHO 3–4 median survival 0.9 months, HR = 5.96 (3.88–9.17) versus WHO 0–1; triple-negative tumors median survival 2.0 months, HR = 1.87 (1.23–2.84) versus Luminal A). Poor performance status and being hospitalized before WBRT were associated with increased ORs of not coming home whereas cohabitation with children at home was protective.

**Conclusion:**

Survival was short following WBRT, and one in four breast cancer patients with brain metastases could never be discharged from hospital. When deciding about WBRT, WHO score, level of care before WBRT, and the patient’s choice of level of care in the end-of-life period should be considered.

**Electronic supplementary material:**

The online version of this article (doi:10.1007/s10549-017-4466-3) contains supplementary material, which is available to authorized users.

## Introduction

Among patients diagnosed with breast cancer, 5–10% have been reported to develop brain metastases [1, 2]. The occurrence of brain metastases in breast cancer patients is believed to have increased over time [3, 4], and HER2-positive and triple-negative breast cancers are associated with an increased risk [5]. The prognosis of breast cancer patients with brain metastases is poor. Overall survival from diagnosis of brain metastases varies from a few months up to a few years in previous studies [5]. Tumor subtype, performance status, age, and the presence or absence of other distant metastases have been identified as prognostic factors [6].

Patients with limited brain metastases may be treated with surgery, and sometimes followed by adjuvant radiation therapy, or with stereotactic radiotherapy [7–9]. Scoring systems have been developed to predict the prognosis for patients with brain metastases such as the Radiation Therapy Oncology Group (RTOG) recursive partitioning analysis (RPA), graded prognostic assessment (GPA), and diagnosis-specific GPA. These prognostic scores [10] provide useful tools in choosing intensive treatments in patients with good prognosis or to identify patients with poor prognosis in order to avoid overtreatment. In recent years, the use of whole brain radiotherapy (WBRT) has decreased due to the development of localized treatment options as well as due to concerns about late toxicity of WBRT. Still, WBRT remains the treatment of choice in patients with poor prognosis, widely spread brain metastases, lower performance status, and uncontrolled systemic disease, the goal being symptom control [11].

If WBRT is initiated in late palliative stages it also affects the level of care in the end-of-life period, including the patient´s possibility to choose level of care. Deaths in hospital are common among cancer patients in Western countries, despite the fact that their home is the most frequent preferred place for dying [12, 13]. Therefore, we studied the survival as well as the level of care in a population-based sample of 241 breast cancer patients with brain metastases before and after WBRT.

## Methods

### Study population and patient characteristics

We identified 281 consecutive patients with intracranial metastases due to breast cancer treated with WBRT at the Karolinska University hospital and Södersjukhuset in Stockholm, Sweden, 1999–2012. Health care in Sweden is tax funded and specialized care in oncology is accessible to all residents. The patients had metastases in the cerebrum, cerebellum, or the leptomeninges. Patients with other cancer diagnoses (*n* = 5), metastases in the scalp only (*n* = 18) or who received radiotherapy in an adjuvant setting following surgery of metastases (*n* = 10) were excluded. The final cohort included 241 patients with breast cancer and brain metastases. Through a detailed review of medical records, we collected data on clinical factors from the primary breast cancer including date of primary diagnosis, tumor subtype, HER2-status, ER-status, TNM-status, and stage. Tumor subtypes was classified as Basal or Triple Negative (ER/PR/HER2 negative), Luminal A (ER/PR positive, HER2 negative), Luminal B (ER/PR/HER2 positive), and HER2 type (ER/PR negative, HER2 positive). We also noted information on neoadjuvant/adjuvant treatment, date and local of first metastases (if not brain), type and number of palliative treatment lines preceding brain metastases (counting each type of administered chemotherapy as one line), the date of diagnosis of brain metastases, and locations of other metastases (visceral, non-visceral or both) at this time. Visceral metastases were defined as lung or liver metastases and non-visceral metastases as bone, skin, or loco-regional spread. We further noted the date of initiation of WBRT, performance status and level of care (home, hospital or hospice) the week before WBRT, administered dose of radiation in Gray (Gy), and family situation (cohabitation and children living at home).

### Outcome information

The main outcome was date of death of any cause. The secondary outcome was if the patient was unable to come home after the treatment.

### Statistical analyses

We estimated the median survival time from the initation of WBRT, with interquartile range. We used Cox proportional hazards models to estimate and compare time to death by patient- and tumor characteristics with hazard ratios (HR) and 95% confidence intervals (CI). In multivariable analyses, we adjusted for age at WBRT in 10-year intervals and calendar period of WBRT in 3-year intervals. In a second multivariable model, we also adjusted for performance status. The proportional hazards assumption was formally tested based on the Schoenfeld residuals obtained from the Cox model and found to be satisfied.

An unconditional logistic regression model was used to identify risk factors for not ever coming home using odds ratios (OR) and 95% CI. This model was first adjusted for age at WBRT in 10-year intervals and calendar period of WBRT in 3 year intervals, and second for performance status.

The SAS software, Version 9.4 of the SAS System for Windows. Copyright © 2002–2012 SAS Institute Inc was used for all analyses.

## Results

### Clinical characteristics of the cohort

The median age at WBRT was 58 years (range 30–88 years) (Table 1). At diagnosis of brain metastases, 60 patients (25%) had brain metastases only and 75% had other distant metastases as well (51 patients (21%) also had visceral metastases, 45 patients (19%) had non-visceral metastases, and 85 patients (35%) had both visceral and non-visceral metastases). Median number of palliative chemotherapy lines before WBRT was 2 (range 0–9). Eighty-two patients (34%) had palliative endocrine therapy before WBRT and 150 patients (62%) had not. Palliative trastuzumab had been given before WBRT to 60 patients (25%).

**Table 1:** Breast cancer patients with brain metastases treated with whole brain radiotherapy, 1999–2012 (total *N* = 241)

Characteristics	Patients	%
At the time of WBRT	*N*	
Age (years)		
30–49	67	28
50–69	137	57
70+	37	15
Median (range)	58 (30–88)	
Calendar year		
1999–2001	38	16
2002–2004	29	12
2005–2007	47	19
2008–2010	74	31
2011–2012	53	22
No of brain metastases		
1–3 (size 5–55 mm)	33	14
4–6	26	11
7–9	11	5
Massive	116	48
Leptomeningeal	53	22
Missing	2	1
Time between diagnosis of breast cancer and WBRT		
0–3 years	94	9
3–6 years	72	30
>6 years	75	31
WBRT dose		
4 Gy × 5	212	88
3 Gy × 10	27	12
2 Gy × 20	1	0
Missing	1	0
WBRT completion		
Yes	228	95
No	13	5
Other metastases		
Only brain	60	25
Brain + visceral^a^	51	21
Brain + non visceral^b^	45	19
Brain + multiple	85	35
WHO performance status score		
0–1	128	53
2	71	29
3–4	41	17
Missing	1	0
Family situation		
Married/cohabitation	96	40
Married/cohabitation, w/children	70	29
Alone, w/children	12	5
Alone	62	26
Missing	1	0
Level of care (1 week before WBRT)		
Home	145	60
Hospital	88	37
Palliative inpatient care	8	3
At primary breast cancer diagnosis		
Stage		
1	44	21
2	108	50
3	40	19
4	22	10
ER		
ER+	135	56
ER−	104	43
Missing ER	2	1
HER2		
HER2+	79	33
HER2−	110	46
Missing HER2	52	22
Subtype		
Luminal A	65	27
Luminal B	36	15
HER2 type	43	18
Triple negative	45	19
Missing	52	22
Palliative treatments before WBRT		
No of palliative chemotherapy regimens		
Median (range)	2 (0–9)	
Missing	5	2
Palliative endocrine therapy		
No	150	62
Yes	82	34
Palliative trastuzumab		
No	172	71
Yes	60	25
Missing	9	4

^a^Lung and/or liver metastases

^b^Skin, loco-regional and/or bone metastases

### Brain metastases and WBRT

At the time of WBRT, about half of the patients (*n* = 116, 48%) had massive metastatic spread to the brain, whereas 33 patients (14%) had 1–3 metastases, 26 (11%) had 4–6 metastases, 11 (5%) had 7–9 metastases, and 53 (22%) had leptomeningeal metastases. Most of the patients (*n* = 212, 88%) were treated with 4 Gy × 5 and 27 (12%) with 3 Gy × 10 and all patients had concomitant steroids. The patients that received 3 Gy × 10 were mostly treated during the early study period (1999–2006). Prior to WBRT, 17 (7%) of the patients had received stereotactic radiotherapy for their brain metastases, 7 (3%) had surgery, and 8 (3%) had both. Thirteen patients (5%) started but could not complete the WBRT. When treatment with WBRT was decided, 129 patients (53%) had WHO performance status score 0–1, whereas 71 (29%) were symptomatic with WHO score 2 and 41 (17%) with WHO score 3–4. In all, 96 (40%) patients were in hospital, whereas 146 (60%) were at home. (Table 1).

### Overall survival

**Fig. 1:**
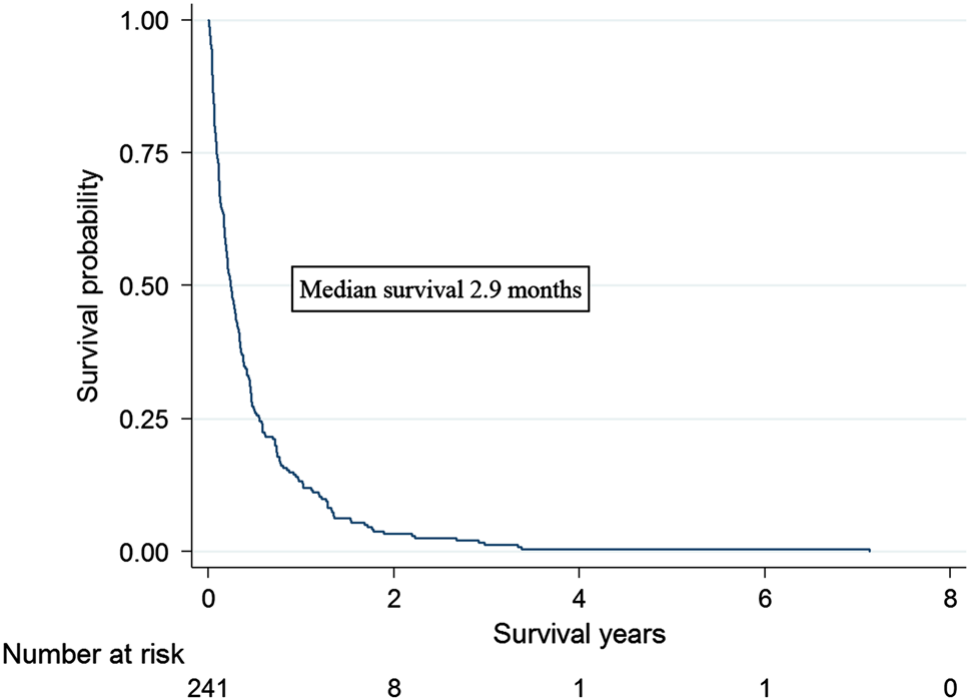
Survival after WBRT for breast cancer patients with brain metastases, in Stockholm, Sweden 1999–2012

Median survival following WBRT was 2.9 months (interquartile range 1.1–6.6 months) (Fig. 1). Performance status was the strongest predictor of survival. Patients with WHO score 2 survived median 2.0 months counting from the first day of WBRT and were at a close to threefold increased rate of death (HR = 2.78, 95% CI 2.01–3.84) and patients with WHO score 3–4 survived less than one (0.9) month corresponding to a sixfold increased rate (HR = 5.96, 3.88–9.17) compared with patients with good performance (WHO score 0–1) (Online Resource 1). Age above 50 years as well as being hospitalized before WBRT was associated with higher mortality before but not after adjustment for performance status (Online Resource 2). Mortality was significantly higher if the patients were treated with WBRT in the first calendar period under study, 1999–2001, compared to later periods.

If the time interval was short (0–3 years) between the primary breast cancer diagnosis and WBRT, mortality following WBRT was significantly higher than if a longer time had passed (3–6 years or more than 6 years). Similarly, mortality was significantly higher if the patients had not been treated with palliative trastuzumab or palliative chemotherapy regimens before WBRT. With respect to the primary breast cancer, triple negative/basal like (HER2 negative, ER negative) and HER2 type (HER2 positive, ER negative) breast cancers were common in this cohort, 45 (19%) and 43 (18%) patients, respectively (Table 1). Whereas primary breast cancer stage and HER2-status did not significantly affect survival following WBRT, ER negative as well as triple-negative tumor status were associated with high mortality (Table 2).

**Table 2:** Survival following whole brain radiotherapy among women with breast cancer and brain metastases, 1999–2012

Characteristics	Median survival months (range)	Adjusted HR^a^ (95% CI)	Adjusted HR^b^ (95% CI)
At the time of WBRT			
Age (years)			
30–49	4.1 (0.2–86.8)	1.0 (ref)	1.0 (ref)
50–69	2.6 (0–40.6)	**1.36 (1.01–1.84)**	1.13 (0.83–1.54)
70+	2.3 (0.1–32.6)	**1.73 (1.15–2.60)**	1.28 (0.84–1.94)
Calendar year			
1999–2001	1.6 (0.17–32.6)	**1.85 (1.20–2.86)**	**1.91 (1.23–2.96)**
2002–2004	4.8 (0.4–86.8)	1.07 (0.67–1.71)	1.06 (0.66–1.70)
2005–2007	3.0 (0–23.0)	1.26 (0.85–1.88)	1.32 (0.88–1.97)
2008–2010	3.1 (0.1–41.1)	1.25 (0.87–1.80)	1.15 (0.80–1.67)
2011–2012	2.8 (0.1–35.4)	1.0 (ref)	1.0 (ref)
No of brain metastases			
1–3 (Size 5–55 mm)	2.5 (0.2–36.2)	1.04 (0.67–1.64)	1.08 (0.68–1.72)
4–6	2.5 (0.3–11.8)	1.33 (0.82–2.15)	1.60 (0.98–2.62)
7–9	3.1 (0.6–12.4)	1.22 (0.63–2.38)	1.46 (0.74–2.88)
Massive	3.0 (0.1–86.8)	0.94 (0.67–1.33)	1.13 (0.80–1.60)
Leptomeningeal	2.5 (0–35.4)	1.0 (ref)	1.0 (ref)
Time between diagnosis and WBRT			
0–3 years	2.1 (0–36.2)	**1.62 (1.17**–**2.24)**	**1.51 (1.09**–**2.10)**
3–6 years	3.5 (0.2–41.1)	**1.47 (1.04**–**2.08)**	1.24 (0.88–1.76)
>6 years	3.6 (0.1–86.8)	1.0 (ref)	1.0 (ref)
WBRT dose			
4 Gy × 5	2.6 (0–86.8)	1.0 (ref)	1.0 (ref)
3 Gy × 10	4.1 (0.2–32.6)	0.73 (0.47–1.13)	0.73 (0.47–1.14)
Other metastases			
Only brain	3.7 (0–86.8)	1.0 (ref)	1.0 (ref)
Brain + visceral^c^	2.8 (0.1–21.6)	1.16 (0.78–1–71)	1.11 (0.74–1.67)
Brain + non visceral^d^	4.1 (0.1–32.6)	0.96 (0.64–1.43)	1.04 (0.70–1.56)
Brain + multiple	2.5 (0.13–41.1)	**1.59 (1.11**–**2.27)**	1.37 (0.96–1.94)
WHO performance status score			
0–1	5.5 (0.4–86.8)	1.0 (ref)	––
2	2.0 (0.1–16.5)	**2.78 (2.01**–**3.84)**	
3–4	0.9 (0–5.5)	**5.96 (3.88**–**9.17)**	
Family situation			
Married/cohabitation	2.4 (0.1–32.6)	1.22 (0.87–1.73)	1.34 (0.95–1.88)
Married/cohabitation w/children	3.2 (0–86.8)	1.11 (0.73–1.70)	1.06 (0.69–1.62)
Alone w/children	8.2 (0.2–41.1)	0.64 (0.33–1.25)	0.85 (0.43–1.68)
Alone	3.1 (0.1–40.6)	1.0 (ref)	1.0 (ref)
Level of care (one week before WBRT)			
Home	4.1 (0.1–86.8)	1.0 (ref)	1.0 (ref)
Hospital	2.0 (0–40.6)	**1.53 (1.16**–**2.04)**	1.03 (0.75–1.40)
Palliative inpatient care	2.5 (0.3–3.7)	**2.60 (1.24**–**5.45)**	1.11 (0.51–2.42)
At primary breast cancer diagnosis			
Stage			
1	4.0 (0.1–86.8)	1.0 (ref)	1.0 (ref)
2	2.5 (0–40.6)	1.21 (0.84–1.73)	1.18 (0.81–1.70)
3	2.6 (0.2–41.1)	1.40 (0.90–2.18)	1.52 (0.97–2.37)
4	1.6 (0.3–21.6)	1.62 (0.96–2.76)	1.38 (0.81–2.37)
ER			
ER+	3.4 (0–86–8)	1.0(ref)	1.0 (ref)
ER−	2.6 (0.2–35.4)	**1.35 (1.03**–**1.77)**	**1.33 (1.01**–**1.77)**
HER2			
HER2+	4.2 (0.1–86.8)	1.0 (ref)	1.0 (ref)
HER2−	2.5 (0–41.1)	1.28 (0.94–1.75)	1.27 (0.92–1.75)
Subtype			
Luminal A	3.5 (0–41.1)	1.0 (ref)	1.0 (ref)
Luminal B	3.9 (0.1–86.8)	0.94 (0.62–1.44)	0.99 (0.64–1.52)
HER2 type	4.1 (0.2–21.6)	1.04 (0.68–1.57)	1.04 (0.68–1.61)
Triple negative	2.0 (0.2–35.4)	**1.87 (1.25**–**2.80)**	**1.87 (1.23**–**2.84)**
Palliative treatments before WBRT			
Palliative trastuzumab			
Yes	5.0 (0.1–86.8)	1.0 (ref)	1.0 (ref)
No	2.4 (0–41.1)	**1.40 (1.03**–**1.90)**	**1.37 (1.01**–**1.86)**
No of palliative chemotherapy regimens			
0	1.6 (0–40.6)	1.0 (ref)	1.0 (ref)
1+	3.5 (0.1–86.8)	**0.61 (0.44**–**0.85)**	**0.65 (0.46**–**0.92)**

Statistically significant results are marked in bold

^a^adjusted for age at WBRT (in 10-year intervals) and calendar period of WBRT (in 3-year intervals)

^b^adjusted for WHO performance status score, age at WBRT (in-10 year intervals) and calendar period of WBRT (in 3-year intervals)

^c^Lung and/or liver metastases

^d^Skin, loco-regional, and/or bone metastases

### Level of care after WBRT

Fifty-seven patients (24%) were never discharged from hospital following WBRT (Table 3). The median survival in this group was 1.1 month (interquartile range 0.5-2.1 months). Among the patients that were hospitalized before WBRT, 45 (47%) did not come home again, whereas this was true for 12 patients (8%) among those who were at home before WBRT (*p* < 0.0001). Among patients with WHO 0–1 before WBRT, 124 (97%) came home again, whereas if WHO was 2, 46 (65%) patients came home, and if the WHO was 3–4, 14 (34%) came home (*p* < 0.0001). In the first logistic regression model adjusting only for age and calendar period, poor performance status was most strongly associated with not coming home (Table 4). Other associated factors included triple-negative tumor status, short duration between primary breast cancer diagnosis and WBRT, few palliative chemotherapy lines, inpatient care before WBRT as well as high age at diagnosis (70+ years, adjusting only for calendar period). With additional adjustment for performance status, hospital or palliative inpatient care the week before WBRT remained significantly associated with not coming home after WBRT. Also, living with a partner and children at home emerged as a protective factor for not coming home (Table 4).

**Table 3:** Patients coming home or not after WBRT by level of care and performance status before

	Ever at home after WBRT		Total
	Yes	No	
Total	184 (76)	57 (24)	241 (100%)
Level of care before WBRT			
Home	133 (92%)	12 (8%)	145 (100%)
Hospital/Palliative inpatient care	51 (53%)	45 (47%)	96 (100%)
WHO performance status score one week before WBRT			
0–1	124 (97%)	4 (3%)	128 (100%)
2	46 (65%)	25 (35%)	71 (100%)
3–4	14 (34%)	27 (66%)	41 (100%)

**Table 4:** The relative risk of not coming home following WBRT by patient and tumor characteristics

Characteristics	*N* (%) of patients not coming home	Adjusted OR^a^ (95% CI)	Adjusted OR^b^ (95% CI)
At the time of WBRT			
Age (years)			
30–49	6 (9)	1.0 (ref)	1.0 (ref)
50–69	37 (27)	**3.76 (1.48**–**9.53)**	2.60 (0.84–8.08)
70+	14 (38)	**6.45 (2.16**–**19.2)**	3.36 (0.91–13.18)
Calendar year			
1999–2006	21 (21)	1.0 (ref)	1.0 (ref)
2007–2012	36 (26)	1.06 (0.55–2.03)	1.07 (0.49–2.38)
No of brain metastases			
1–3 (Size 5–55 mm)	8 (24)	1.0 (ref)	1.0 (ref)
4–6	8 (30)	1.72 (0.48–6.12)	2.44 (0.50–12.1)
7–9	2 (18)	0.73 (0.13–4.56)	1.40 (0.18–11.0)
Massive	26 (22)	1.19 (0.43–3.27)	1.57 (0.47–5.18)
Leptomeningeal	13 (25)	1.26 (0.42–3.80)	1.21 (0.33–4.37)
Time between diagnosis and WBRT			
0–3 years	30 (32)	1.0 (ref)	1.0 (ref)
3–6 years	12 (17)	0.45 (0.20–1.00)	0.54 (0.20–1.44)
>6 years	15 (20)	**0.43 (0.20**–**0.93)**	0.47 (0.19–1.17)
WBRT dose			
4 Gy × 5	51 (24)	1.0 (ref)	1.0 (ref)
3 Gy × 10	6 (22)	1.19 (0.43–2.30)	1.54 (0.43–5.46)
Other metastases			
Only brain	14 (23)	1.0 (ref)	1.0 (ref)
Brain + visceral^c^	9 (18)	0.75 (0.27–2.03)	0.66 (0.20–2.21)
Brain + non visceral^d^	11 (24)	1.20 (0.46–3.11)	1.82 (0.53–6.24)
Brain + multiple	23 (27)	1.85 (0.80–4.26)	1.24 (0.46–3.34)
WHO performance status score			
0–1	4 (3)	1.0 (ref)	–
2	25 (35)	**16.9 (5.39**–**53.1)**	–
3–4	27 (66)	**54.5 (15.7**–**189)**	–
Family situation			
Married/cohabitation	25 (26)	0.63 (0.30–1–31)	0.54 (0.22–1.37)
Married/cohabitation w/children	8 (11)	0.38 (0.13–1–09)	**0.21 (0.06**–**0.78)**
Alone w/children	1(8)	0.22 (0.02–1.97)	1.27 (0.11–14.97)
Alone	23 (37)	1.0 (ref)	1.0 (ref)
Level of care (1 week before WBRT)			
Home	12 (8)	1.0 (ref)	1.0 (ref)
Hospital	40 (45)	**10.2 (4.63**–**22.4)**	**5.20 (2.12**–**12.7)**
Palliative inpatient care	4 (63)	**21.2 (4.03**–**111)**	5.27 (0.89–31.06)
At primary breast cancer diagnosis			
ER			
ER+	31 (23)	1.0 (ref)	1.0 (ref)
ER−	26 (25)	1.11 (0.57–2.14)	1.19 (0.52–2.74)
HER2			
HER2+	14 (18)	1.0 (ref)	1.0 (ref)
HER2−	31 (28)	2.04 (0.93–4.49)	1.97 (0.70–5.54)
Subtype			
Luminal A	12 (18)	1.0 (ref)	1.0 (ref)
Luminal B	10 (28)	1.69 (0.60–4.80)	2.66 (0.66–10.72)
HER2 type	4 (9)	0.36 (0.10–1.30)	0.39 (0.09–1.80)
Triple negative	19 (42)	**2.57 (1.02**–**6.50)**	2.85 (0.89–9.15)
Palliative treatments before WBRT			
Palliative trastuzumab			
Yes	10 (17)	0.63 (0.28–1.40)	0.68 (0.25–1.90)
No	46 (27)	1.0 (ref)	1.0 (ref)
No of palliative chemotherapy regimens			
0	22 (42)	1.0 (ref)	1.0 (ref)
1+	35 (19)	**0.42 (20**–**0.85)**	0.50 (0.21–1.19)

Statistically significant results are marked in bold

^a^Adjusted for age at WBRT (in 10-year intervals) and calendar period of WBRT (in 3-year intervals)

^b^Adjusted for WHO performance status score, age at WBRT (in 10-year intervals) and calendar period of WBRT (in 3-year intervals

^c^Lung and/or liver metastases

^d^Skin, loco-regional and/or bone metastases

## Discussion

In this population-based study of consecutive breast cancer patients with brain metastases treated with WBRT, median survival was short, less than 3 months counting from the start of WBRT, and one in four patients could not be discharged from hospital following radiotherapy. As expected, we observed a significant association between triple-negative tumors, poor performance status, and short survival. Patients with WHO score 3–4 had a short median survival of less than 1 month and two-thirds of these patients were not able to come home again. The risk of not coming home again was affected not only by performance status, but also by the level of care the week before treatment with WBRT and to some extent by family situation (if the patient was living with a partner with children at home or not). Given the delayed effect of radiotherapy, our results support that hospitalized patients with poor performance status and short expected survival may benefit more from abstaining from WBRT rather than receiving it.

Patients with limited brain metastases may be treated with surgery, sometimes followed by adjuvant radiation therapy or with stereotactic radiotherapy [7–9]. For patients with a single, large metastasis in a surgically accessible location, resection may still be the best choice for control of symptoms and prognosis. Surgery is also used in some patients with a limited number of metastases. Randomized clinical trials that have compared surgery plus WBRT to WBRT alone in patients with a single brain metastasis, have shown a survival benefit for the combined approach [14–16]. Stereotactic radiosurgery is recommended to patients who are inoperable with 4–5 metastases at most (of diameter 3 cm or less) and with few or no symptoms. In light of these developments of localized treatment, the use of WBRT as only treatment has decreased. Still, WBRT remains the treatment of choice in patients with poor prognosis, widely spread brain metastases, lower performance status, and uncontrolled systemic disease, the goal being symptom control and improvement of neurological deficits [11]. At the same time, the risk of overtreatment is particularly present among these poor prognosis patients [10]. Earlier studies have shown that 50–80% of the patients respond with improvement of neurological symptoms after WBRT [17–20]; however, improvements may be first noted a few days or up to a few weeks after WBRT [21]. Also, the duration of an improvement may be short. In one previous study, the median duration of effect was observed to be 3.7 month (range 0.4–11.8 months) [22].

To our knowledge, this is the first study of level of care in brain metastasis patients treated with WBRT. Level of care following treatment is expected to vary not only by patient and disease characteristics and survival but also external factors such as social situation and access to advanced palliative care at home. In spite of easy access to palliative home care in the Stockholm area, we were surprised to note that as many as 25% of all patients in our study could not be discharged from hospital following WBRT. Median survival in our cohort was not shorter (2.9 months from the start of WBRT) than previously reported. Patients with brain metastases of any cancer have been described to have a median survival of 3 to 6 months following diagnosis of brain metastases in two single center studies [23, 24]. In a recent observational study from two university specialist care centers, local treatment with surgery or stereotactic radiosurgery with or without WBRT was compared with WBRT alone in breast cancer patients with brain metastases (single or multiple). Among the 116 patients in the study, 50 patients were treated with WBRT alone, and the overall survival was median 5.7 months after brain metastases diagnosis in this group (95% CI 0.5–11 months) [25]. Since we counted survival from the start of WBRT it is expected that we report shorter survival. The patients in our study lived in the Stockholm area, geographically close to the hospital radiotherapy departments. The relatively easy access could have resulted in a broader use of WBRT compared to other centers, i.e., including patients who might have been judged unfit under different conditions, potentially lowering the survival in our cohort. On the other hand, previous specialist single center studies may have included more selected patient groups in comparison to our population-based assessment.

As expected, poor performance status and a triple-negative primary breast cancer were associated with dismal outcome following WBRT. Within the breast cancer specific scores (GPA) high age, poor performance status, Karnofsky index less than 50, high number of brain metastases, and a triple-negative primary tumor predict short survival [6, 26]. Level of care before treatment with WBRT was associated with survival when adjusted for age, but not with additional adjustment for performance status. However, the odds ratio for not coming home after WBRT was still significantly higher after adjustment for age and performance status.

Among all breast cancer patients diagnosed in Stockholm in 2014, triple-negative breast cancers constituted 8.2%, the HER2 type 13.8%, and the Luminal A and B types together represented 78.1% [27]. In this study, triple-negative tumors (19%) and HER2 type tumors (18%) cancers were more common.

For breast cancer patients with brain metastases and unfavorable prognostic characteristics as outlined above it would likely be more appropriate in many cases to recommend best supportive care including steroids for symptom control instead of WBRT and discuss goals of care with the patient. Time spent on hospital stays for WBRT would then be spared and treatment side effects would be avoided for patients in late palliative stages if withheld from WBRT. In Western countries deaths in hospital are common among cancer patients, despite the fact that their home is the most frequent preferred place for dying [12, 13]. In an Italian study, researchers sampled 2000 patients who died due to cancer from March 2002 until June 2003. The non-professional caregiver, defined as the person closest to the patient and best informed about the patient’s situation during the last three months of life, was identified and interviewed. The caregivers reported that home was the most preferred place of death for 94% of the cancer patients, with a range between 90 and 99% within the country. In a recent systematic review of worldwide studies of preferred place of death among cancer patients a preference of home deaths was found among 59.9% (range 39.7–100%) on average across all studies. The preferred and actual death places among these studies differed significantly (*p* < 0.05). The lack of conformance between the patients preferred place of death and where they actually die seems to be the same worldwide [13]. In our study we found that patients living with a partner and children at home were discharged from hospital more often than patients in other family situations. These patients may have a stronger desire to come home and a higher ability to communicate their wishes to health professionals together with family members. A Chinese study from 2015 reported that cancer patients, who live with relatives more often prefer home as death place [28]. Our findings support that when patients with brain metastases due to breast cancer are in late palliative stages, discussions and considerations about the patient’s choice of level of care in the end-of-life period should take place.

The strengths of our study include the population-based identification of all consecutively treated patients in the Stockholm area and the use of prospectively recorded exposure and outcome data from medical records. A limitation was the relatively small number of patients, leading to low precision in some analyses and the sometimes indirect estimation of variables such as WHO performance status score based on the medical record notes.

To summarize, in this clinical study of consecutively treated patients, one in four breast cancer patients with brain metastases could not be discharged from hospital following WBRT. These results suggest some overtreatment of WBRT in late palliative stages and encourages use of existing scores such as the breast cancer specific GPA to help predict prognosis and choose the most optimal treatment and care together with the patient. For patients with poor performance status requiring hospital care and with unfavorable breast cancer subtypes, best supportive care including steroids for symptom control should be considered to avoid hospital stays in the end-of life period. When deciding about WBRT, the patient´s performance status, level of care before WBRT, and importantly also the patient´s choice of care in the end-of-life period should be taken into consideration.

## Electronic supplementary material

Below is the link to the electronic supplementary material.

Supplementary material 1 (PDF 48 kb)

## Abbreviations

WBRT: Whole Brain Radiotherapy
WHO: World Health Organization
