# Supplementary material for: Survival and level of care among breast cancer patients with brain metastases treated with whole brain radiotherapy
Source: Breast Cancer Res Treat. 2017 Aug 22;166(3):887–96. doi: 10.1007/s10549-017-4466-3 (PMC5680371; doi:10.1007/s10549-017-4466-3)

# SURVIVAL AND LEVEL OF CARE AMONG BREAST CANCER PATIENTS WITH BRAIN METASTASES TREATED WITH WHOLE BRAIN RADIOTHERAPY

**Breast cancer research and treatment**

Gabriella Frisk[1], Beatrice Tinge[1], Sara Ekberg[1], Sandra Eloranta[1], L Magnus Bäcklund[2], Elisabet Lidbrink[3], Karin E Smedby[1]

[1]Department of Medicine Solna, Clinical Epidemiology Unit, Karolinska Institute Solna, SE-171 76 Stockholm, Sweden

[2]Department of Medicine Solna, Unit for Experimental cardiovascular research, Karolinska Institute Solna, SE-171 76 Stockholm, Sweden

[3]Department of Oncology-Pathology, Karolinska Institute, Karolinska University Hospital Solna, SE-171 76 Stockholm, Sweden

**Corresponding author**: Gabriella Frisk

E-mail: gabriella.frisk@ki.se

**Online Resource 1. Survival after WBRT among breast cancer patients with brain metastases in relation to performance status**

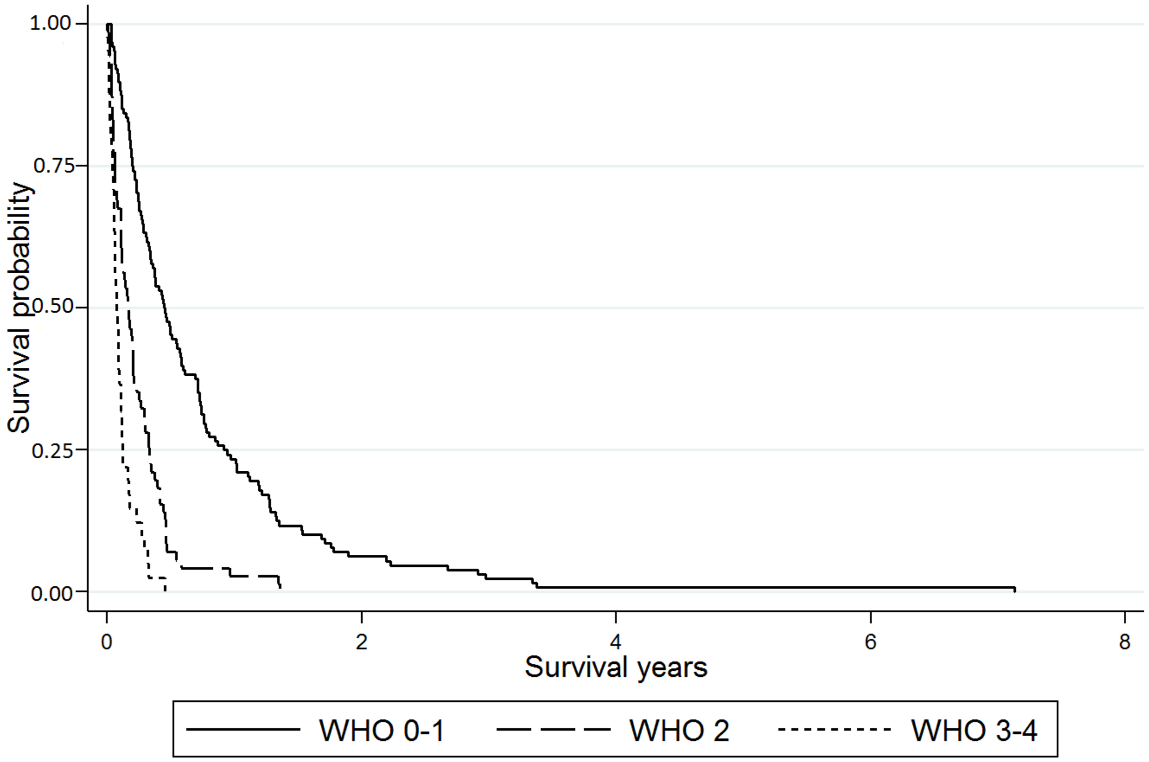

**Online Resource 2. Survival after WBRT among breast cancer patients with brain metastases, relation to level of care**

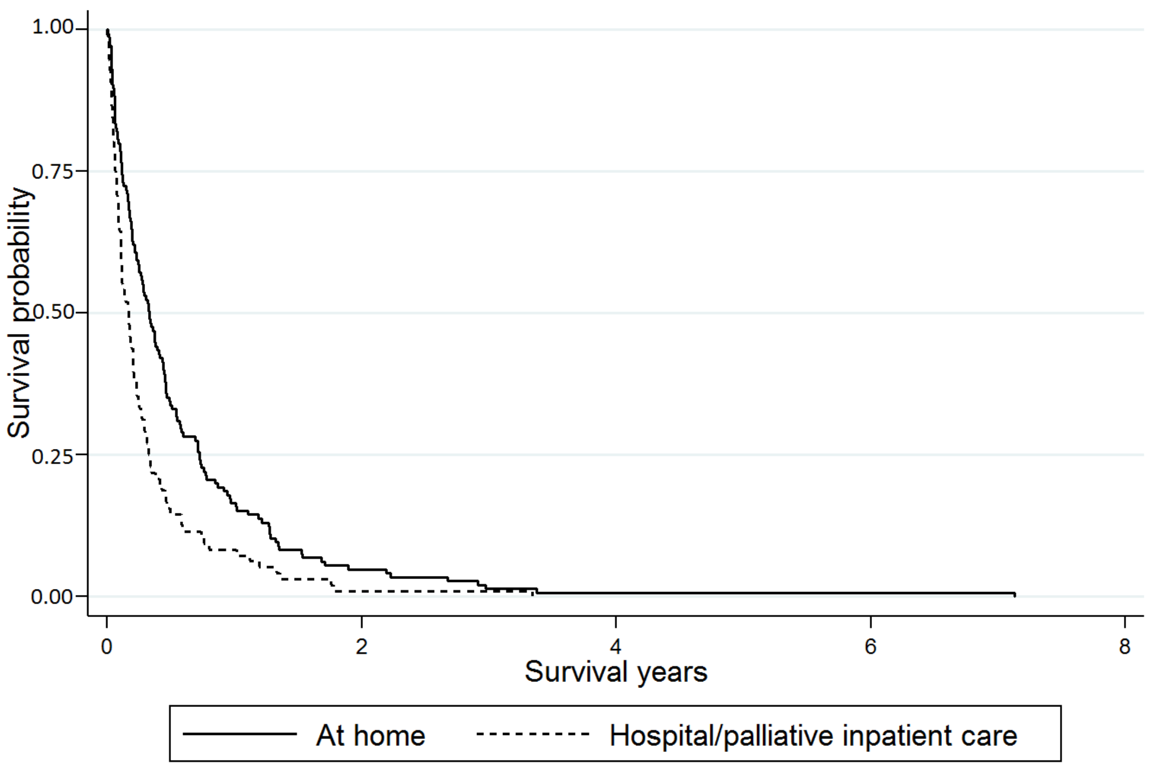

Supplement: Supplementary file 1 — Supplementary material 1 (PDF 48 kb) [file 10549_2017_4466_MOESM1_ESM.pdf]
